# Longitudinal tumor-informed ctDNA monitoring for recurrence and treatment response in melanoma

**DOI:** 10.1186/s12885-026-15931-5

**Published:** 2026-04-02

**Authors:** Olivia Sleeper, Justin Fortino, Nam Woo Cho, Adil I. Daud, Katy K. Tsai, James C. Lee, Michael Alvarado, Ajay V. Maker, Lauren Liu, Christine Kim, Jason W. Chan

**Affiliations:** 1 Department of Radiation Oncology, University of California, San Francisco, 1825 Fourth St. First Floor, Room L1101, San Francisco, CA 94158 USA; 2 Department of Medicine, University of California, San Francisco, San Francisco, CA USA; 3 Department of Surgery, University of California, San Francisco, San Francisco, CA USA; 4 UCSF Health, San Francisco, CA USA

**Keywords:** ctDNA, Melanoma, Minimal residual disease

## Abstract

**Background:**

Early detection of melanoma recurrence and progression remains a clinical challenge, which relies on physical examination and imaging. Circulating tumor DNA offers a noninvasive, dynamic biomarker that may identify molecular recurrence before clinical progression. We aimed to evaluate the prognostic and predictive value of longitudinal, tumor-informed ctDNA testing across multiple melanoma care settings.

**Methods:**

We retrospectively analyzed 56 consecutive melanoma patients who underwent serial ctDNA testing using a tumor-informed assay (Signatera™) at a single institution. Fifty-six patients with evaluable ctDNA results were stratified into three cohorts based on the treatment context at the time of ctDNA testing: (A) no active treatment (*n* = 20), (B) adjuvant therapy (*n* = 14), and (C) active treatment for known disease (*n* = 22). We evaluated disease-free survival (DFS), overall survival (OS), and longitudinal ctDNA dynamics in relation to clinical outcomes.

**Results:**

Median clinical follow-up was 48.0 (IQR 24.7–94.1) months. In Cohorts A and B (HR 12.3, 95% CI 1.1–1138.6), detectable ctDNA at any timepoint was significantly associated with inferior DFS. Conversely, 90% of patients in Cohorts A and B combined with undetectable ctDNA remained recurrence-free. In Cohort C, rising or persistently positive ctDNA was seen in 81.8% of patients who progressed. Longitudinal ctDNA trends were more informative than isolated timepoints, with increases preceding radiologic progression by a median of 11.4 (IQR 5.0–13.1) months.

**Conclusions and relevance:**

Tumor-informed ctDNA testing offers clinically meaningful insight across melanoma care settings. Rising or positive ctDNA frequently anticipates disease progression, supporting its use for MRD surveillance and treatment response monitoring in dermatology and oncology practice.

## Introduction

Predicting recurrence and treatment failure in melanoma remains a major clinical challenge. Current surveillance strategies, including routine imaging and physical exams, often fail to detect disease recurrence and progression in a timely manner. There is an urgent need for sensitive, noninvasive biomarkers in melanoma that can dynamically reflect tumor burden, guide treatment decisions, and personalize patient monitoring.

Circulating tumor DNA (ctDNA), comprising tumor-derived DNA fragments in the bloodstream, has emerged as a promising biomarker for detecting MRD and monitoring treatment response across solid tumors, including melanoma [1–4]. In melanoma, ctDNA monitoring has been studied using droplet digital PCR (ddPCR) for specific mutations such as *BRAF V600E/K* and *NRAS Q61* or next-generation sequencing (NGS)-based approaches for broader genomic coverage [5–7]. Serial ctDNA measurements during therapy correlate with disease status and prognosis, with rising levels often preceding radiographic progression [4, 8, 9]. In the adjuvant setting, ctDNA monitoring can detect molecular relapse months before clinical or radiographic recurrence, potentially enabling earlier intervention [7, 9].

In this study, we report a single-institution experience using longitudinal, tumor-informed ctDNA testing in a real-world cohort of melanoma patients across three clinically distinct contexts: post-treatment surveillance, adjuvant therapy, and active systemic treatment. By examining longitudinal ctDNA dynamics alongside clinical and radiographic outcomes, we aim to define its role in MRD detection, therapeutic monitoring, and guiding care decisions in dermatologic oncology.

## Methods

### Study design and participants

Patients with melanoma who underwent ctDNA testing at a single institution from January 2021 to June 2024 were included. Clinical data, treatment history, radiographic findings, and clinical outcomes were extracted from medical records. Patients were stratified into three cohorts based on treatment context at the time of ctDNA testing: Cohort A: surveillance in the absence of clinically-detectable disease; Cohort B: receiving adjuvant therapy in the absence of clinically-detectable disease; Cohort C: Receiving primary systemic therapy for clinically detectable disease.

### ctDNA testing

All patients underwent personalized ctDNA testing with the Signatera™ assay (Natera Inc), which uses whole-exome sequencing of tumor and matched normal DNA to track 16 tumor-specific variants in plasma. Results were classified per manufacturer thresholds of mean tumor molecules as negative (0 MTM/mL), indeterminate or “in sensitivity range” (0.01–0.3 MTM/mL), or positive (> 0.3 MTM/mL).

### Statistical analysis

Disease-Free Survival (DFS) in Cohorts A and B and Overall Survival (OS) in Cohort C were estimated using Kaplan-Meier analysis. Associations between ctDNA status and clinical outcomes were assessed using log-rank tests. Longitudinal ctDNA dynamics were visually analyzed relative to treatment course and radiographic progression. R version 4.5.1 was used for all analyses.

## Results

### Baseline patient and tumor characteristics by cohort

**Fig. 1:**
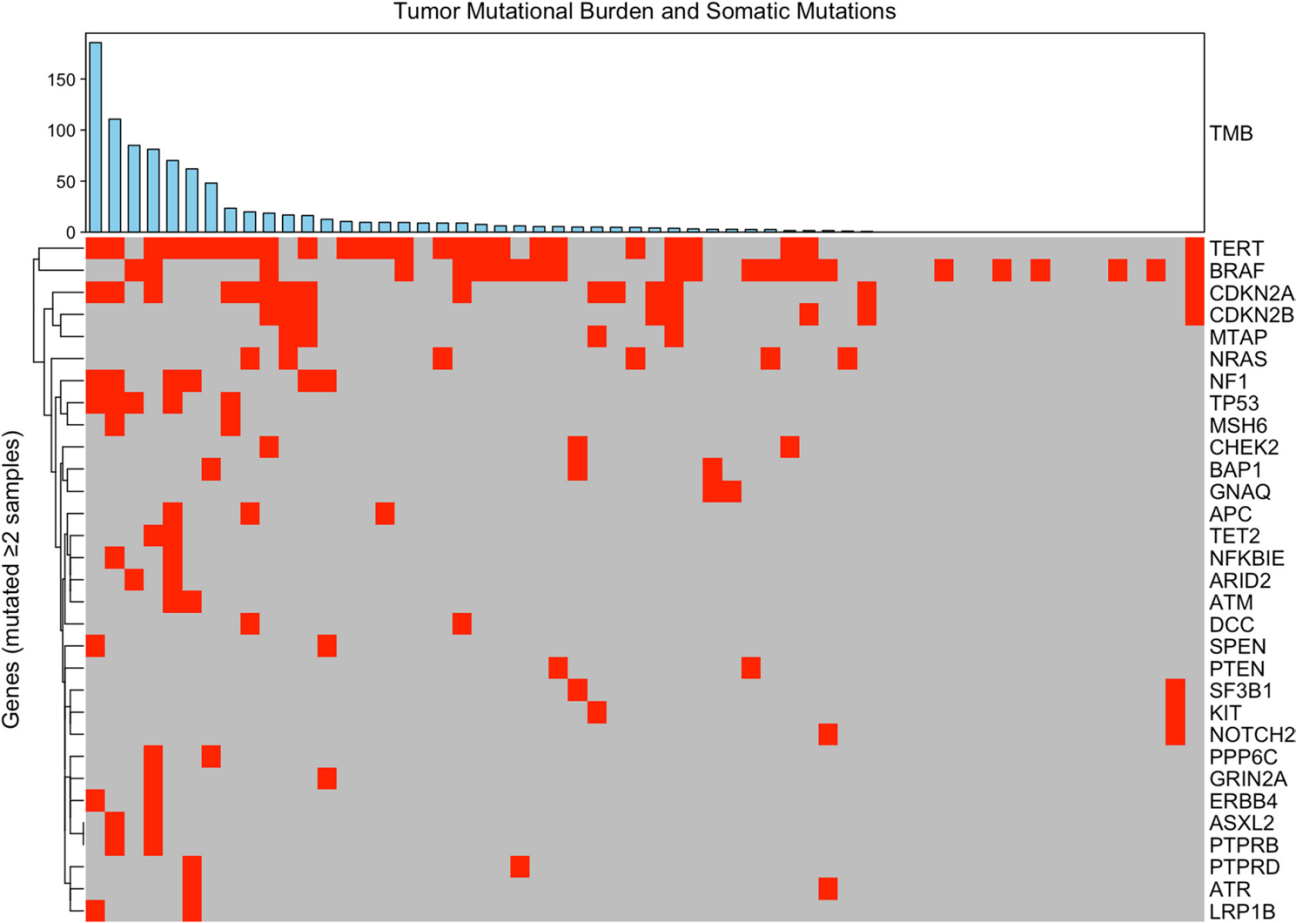
Tumor Mutation Burden, PD-L1, and Somatic Mutations. Tumor mutational burden (TMB, top) and PD-L1 expression (middle) ordered by decreasing TMB. The most frequently mutated genes (≥ 2 patients) from whole exome and transcriptome sequencing are shown below

Fifty-six patients with melanoma underwent longitudinal, tumor-informed ctDNA testing, and had evaluable ctDNA results for analysis. Patients were stratified into three clinically defined cohorts: Cohort A (*n* = 20) included individuals not receiving active treatment, typically under surveillance after definitive therapy; Cohort B (*n* = 14) included patients receiving adjuvant systemic therapy but without clinically-detected disease; and Cohort C (*n* = 22) included patients undergoing treatment for active metastatic or unresectable disease. For each cohort, patient and tumor characteristics were assessed, including AJCC stage, ulceration status, *BRAF* mutation status, LDH level, melanoma subtype, treatment type, and sites of metastasis. Clinicopathologic features at the time of ctDNA testing initiation are summarized in Table 1. Tumor mutational burden and tumor somatic mutation profiles are presented in Fig. 1.

**Table 1:** Clinicopathologic and Treatment Characteristics of Melanoma Patients by Clinical Cohort at ctDNA Testing Initiation. Baseline demographic, clinical, and treatment characteristics of 56 melanoma patients undergoing ctDNA testing: Cohort A (no active treatment, n = 20), Cohort B (adjuvant therapy, n = 14), and Cohort C (treatment for active disease, n = 22)

Characteristic	Cohort A (N=20)	Cohort B (N=14)	Cohort C (N=22)
Clinical Stage			
I	1 (5.0)	0 (0)	0 (0)
II	2 (10.0)	1 (7.1)	0 (0)
III	9 (45.0)	4 (28.6)	6 (27.3)
IV	8 (40.0)	9 (64.3)	16 (72.7)
Ulceration			
Absent	11 (54.0)	9 (64.3)	13 (59.1)
Present	9 (45.0)	5 (35.7)	9 (40.9)
BRAF Status			
Wildtype	12 (60.0)	9 (64.3)	13 (59.1)
Mutated	8 (40.0)	5 (35.7)	9 (40.9)
LDH Level			
Normal	16 (80.0)	12 (85.7)	13 (59.1)
Elevated (>280 U/L)	1 (5.0)	0 (0)	5 (22.7)
Unknown	3 (15.0)	2 (14.3)	4 (18.2)
Subtype			
Cutaneous	18 (90.0)	14 (100)	16 (72.7)
Mucosal	1 (5.0)	0 (0)	5 (22.7)
Ocular	1 (5.0)	0 (0)	1 (4.5)
Treatment			
PD-1 (Nivolumab, Pembrolizumab)	0 (0)	12 (85.7)	14 (63.6)
BRAF/MEK (Dabrafenib/Trametinib, Encorafenib/Binimetinib)	0 (0)	1 (7.1)	0 (0)
PD-1/CTLA-4 (Ipilimumab/Nivolumab)	0 (0)	1 (7.1)	8 (36.4)

### Associations between ctDNA status with recurrence and overall survival

**Fig. 2:**
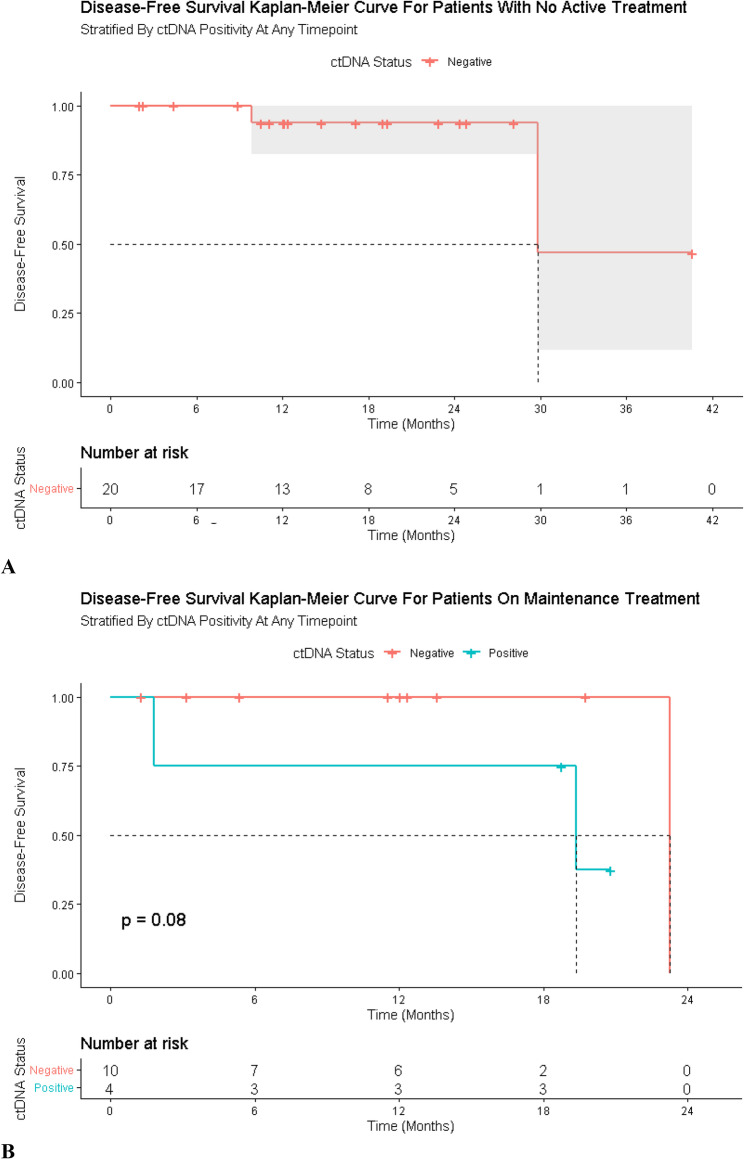
Disease-Free Survival in Patients Undergoing Observation, Stratified by ctDNA Status. Disease-free survival for patients with melanoma without clinically-detected disease and not receiving treatment (**A**) and, receiving adjuvant therapy **B**

Patients with persistently negative ctDNA were associated with prolonged disease-free intervals. In Cohorts A and B combined, 82.35% (28/34) remained recurrence-free (Table 2). In Cohort A, patients under surveillance with undetectable ctDNA remained recurrence-free in most cases (Fig. 2A). In Cohort B, ctDNA negativity during adjuvant therapy was associated with lower relapse risk.

**Table 2:** Summary Statistics of ctDNA Testing by Clinical Cohort. Average number of tests, ctDNA positivity rate, and recurrence rate of 56 melanoma patients undergoing ctDNA testing: Cohort A (no active treatment, n = 20), Cohort B (adjuvant therapy, n = 14), and Cohort C (treatment for active disease, n = 22)

	Cohort A	Cohort B	Cohort C
Median (IQR) Tests Per Patient	6 (3.8–9.0)	9 (3.8–12.3)	10 (3.8–12.8)
Number (Rate) of Positive ctDNA Results	0 (0.0%)	4 (28.6%)	18 (81.8%)
Number (Rate) of Recurrence	3 (15.0%)	3 (21.4%)	11 (50.0%)
Primary Recurrence	0 (0.0%)	1 (7.1%)	0 (0.0%)
Nodal Recurrence	2 (10.0%)	2 (14.3%)	7 (31.8%)
Distant Recurrence	3 (15.0%)	3 (21.4%)	10 (45.5%)
Brain Metastases	0 (0.0%)	0 (0.0%)	7 (31.8%)
Lung Metastases	3 (15.0%)	2 (14.3%)	5 (22.7%)
Liver Metastases	1 (5.0%)	2 (14.3%)	7 (31.8%)
Other Distant Metastases	2 (10.0%)	2 (14.3%)	10 (45.5%)

**Fig. 3:**
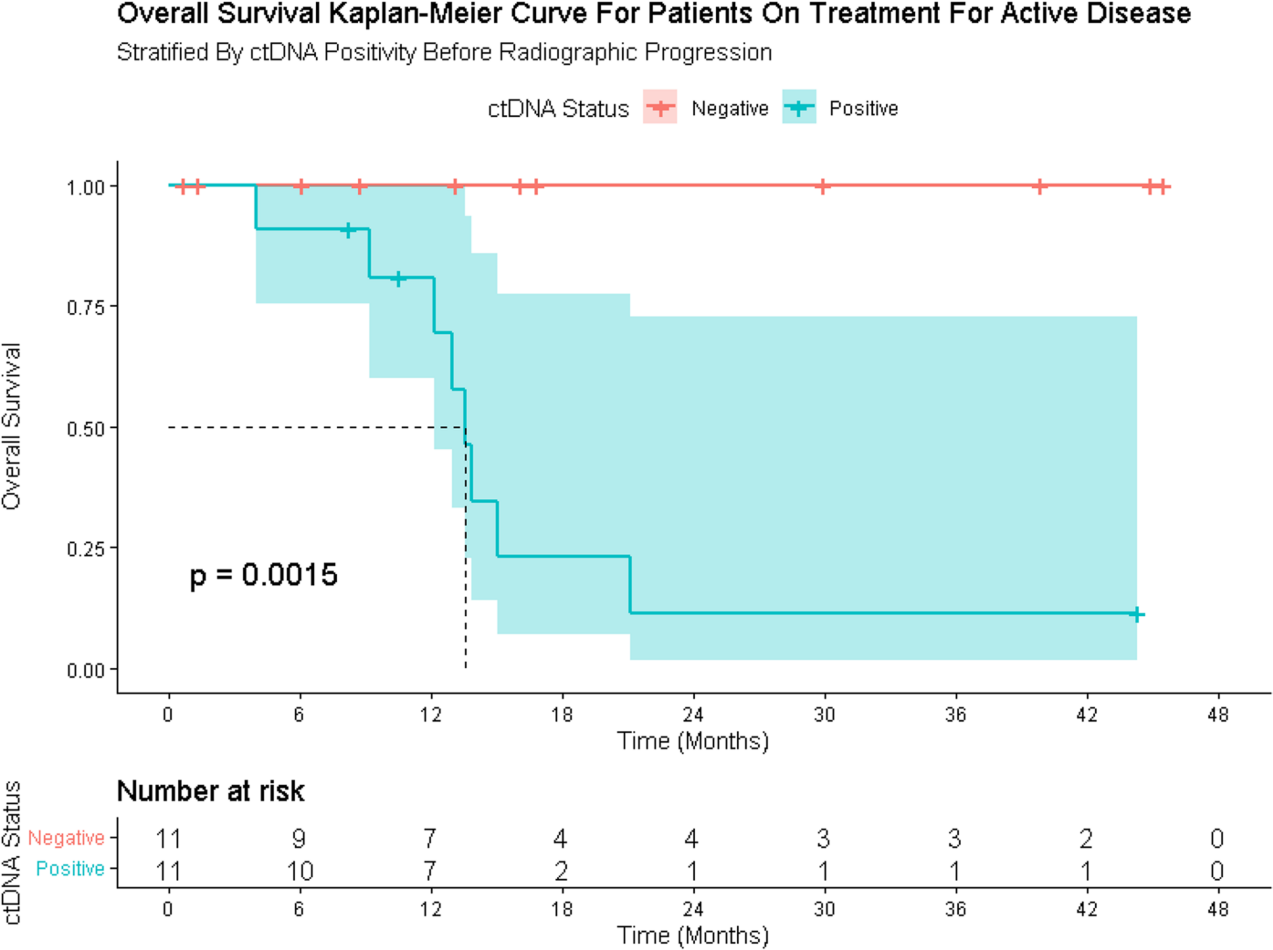
Overall survival in patients receiving therapy for active disease, stratified by ctDNA Status. Overall survival (OS) stratified by ctDNA positivity at any timepoint for patients receiving treatment for clinically apparent disease (Cohort C)

Conversely, in Cohort C, persistent or rising ctDNA was frequently observed in patients who eventually progressed. In the subset of patients in Cohort C who had a positive ctDNA test and had two tests within three months of progression, 100% of patients (6/6) experienced a rise in ctDNA levels or a continuation of ctDNA positivity three months before their progression. The presence of ctDNA positivity at any timepoint correlated with poorer survival (Fig. 3).

### Lead time to radiographic progression

**Fig. 4:**
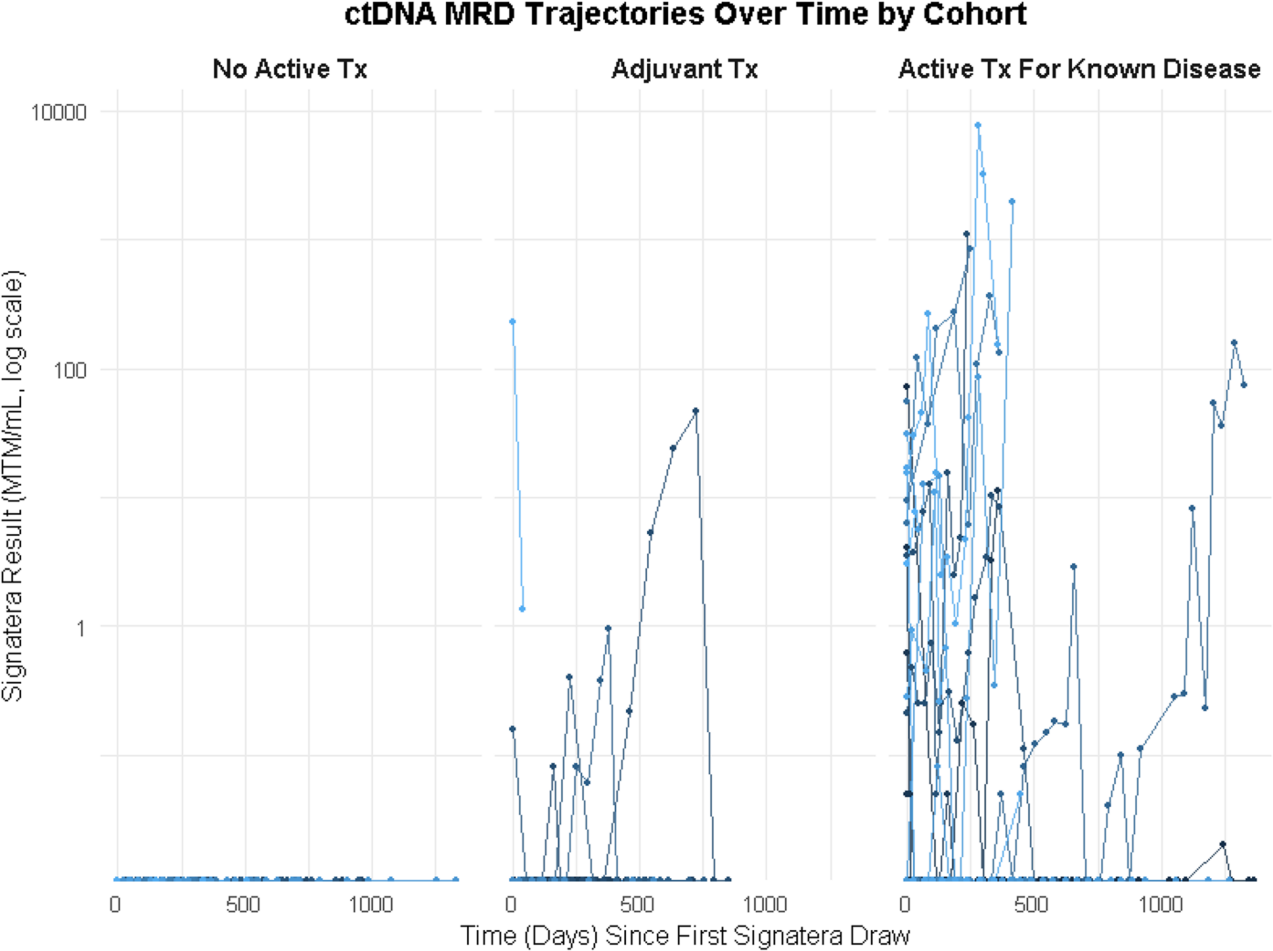
Longitudinal MRD Dynamics by clinical cohort. ctDNA dynamics over time for all patients separated by clinical cohort. Patients on surveillance or adjuvant therapy showed ctDNA clearance or persistently undetectable values in most cases. In contrast, patients on active treatment for known disease exhibited persistently high or fluctuating ctDNA levels

**Fig. 5:**
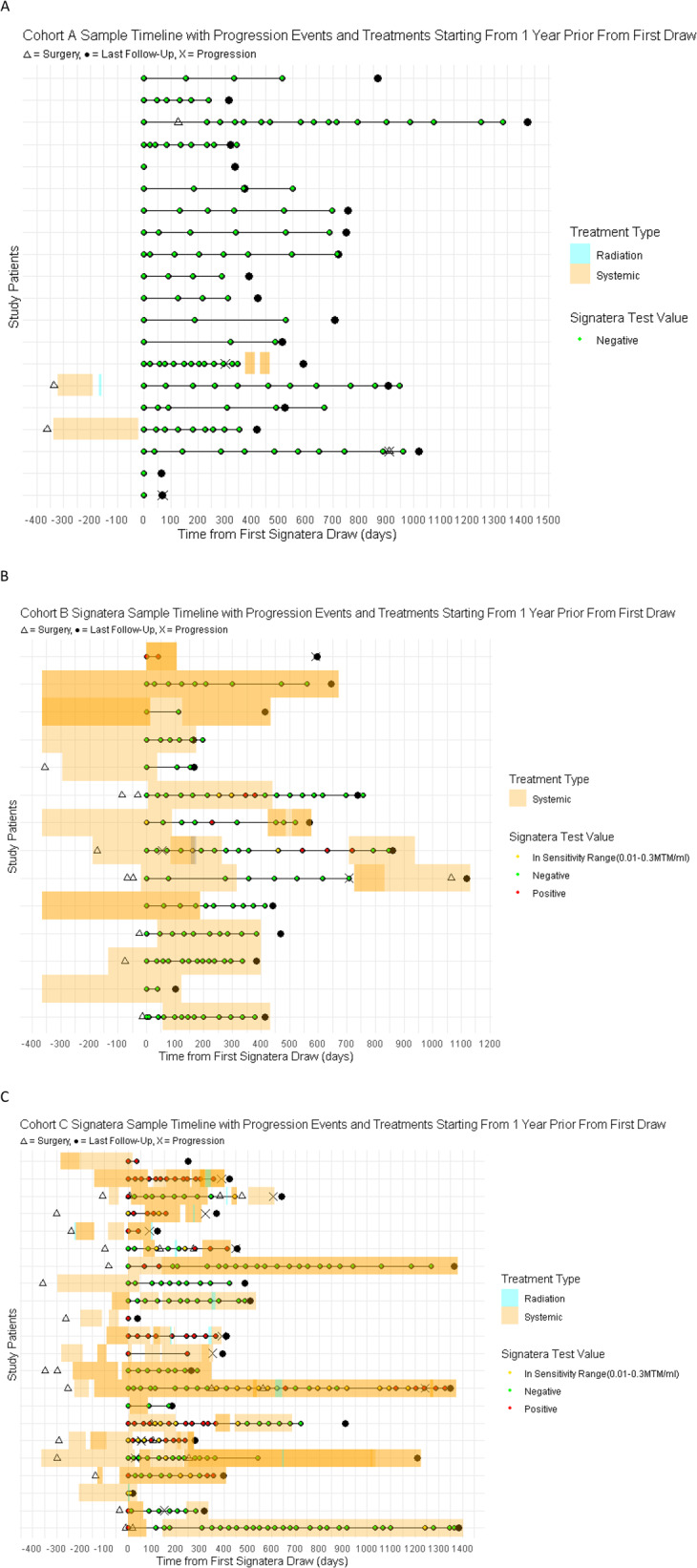
Timelines of Treatments, ctDNA Testing, and Clinical Events by Cohort. Individual patient timelines from the first ctDNA test. Radiation (blue bars), systemic therapy (orange bars), surgeries (triangles), progression events (X), and last follow-up (diamonds) are annotated. Each ctDNA result is shown by color-coded dot: green (negative), red (positive), yellow (in sensitivity range). Treatments more than 1 year prior from their first Signatera draw but were excluded for visual clarity

Serial ctDNA measurements revealed distinct temporal patterns by cohort (Fig. 4). In Cohorts A and B, ctDNA was either undetectable throughout or showed clearance after treatment initiation, correlating with durable remission. In contrast, Cohort C exhibited more variable ctDNA trajectories, including persistent positivity, transient reductions, and rebound increases. These patterns aligned closely with radiographic progression or treatment failure (Fig. 5). In Cohort C, ctDNA dynamics often anticipated clinical progression with a median lead time of 11.42 months (IQR 5.03–13.13). Conversely, patients with durable treatment responses had sustained ctDNA clearance.

## Discussions

In this single-institution, real-world cohort spanning the melanoma disease continuum, longitudinal tumor-informed ctDNA analysis provided prognostic and predictive information beyond standard imaging. In post-treatment surveillance (Cohort A) and adjuvant treatment (Cohort B) settings, persistently negative ctDNA identified patients with durable remission (90% recurrence-free at last follow-up), while conversion to positivity flagged high likelihood of recurrence. These findings are consistent with multiple studies showing ctDNA positivity predicts recurrence in melanoma after definitive therapy. In the COMBI-AD trial, post-resection ctDNA positivity (detected in 13% of stage III melanoma patients) identified a high-risk group with significantly shorter recurrence-free survival and distant metastasis-free survival [5]. Similarly, Lee et al. found that detectable post-surgery ctDNA was associated with decreased disease-free (HR 3.12) and distant metastasis-free intervals (HR 3.22) in high-risk stage II/III melanoma patients. Furthermore, Tan et al. found that 90% of patients with ctDNA detection in post-surgical samples who did not receive adjuvant therapy relapsed at a median follow-up of 20 months [10]. In CheckMate 915, patients with positive post-surgical ctDNA who cleared ctDNA by week 7 of adjuvant therapy had markedly better recurrence-free survival (median not reached) compared to those with persistently positive ctDNA (median 3.35 months).

On-treatment ctDNA dynamics distinguished responders from non-responders with substantial lead time over radiographic progression. Among our patients with unresectable or metastatic melanoma receiving systemic therapy (Cohort C), rising ctDNA levels frequently preceded radiographically confirmed progression or treatment resistance, with a median lead time of 11.42 months. Similarly, Eroglu et al. reported that in unresectable stage III/IV patients receiving immune checkpoint inhibitors, all ctDNA-negative patients remained progression-free for a median follow-up of 14.7 months whereas ct-DNA positive patients experienced disease progression [4]. In uveal melanoma, Rodrigues et al. reported that baseline ctDNA detection and longitudinal ctDNA measurements with ddPCR were associated with overall survival in patients with metastatic disease [11]. Detecting molecular residual disease (MRD) in melanoma prior to clinical progression may allow for earlier therapeutic intervention and improve patient outcomes.

Limitations of this study include its retrospective design, single-center setting, and small sample size. Larger, prospective studies are needed to validate thresholds for clinical intervention based on ctDNA trends and to better define optimal ctDNA testing intervals.

## Conclusions

Serial tumor-informed ctDNA analysis is a promising biomarker to guide surveillance and treatment in melanoma. Incorporating MRD-based monitoring into standard of care pathways may better refine risk stratification and personalize melanoma management.

## Data Availability

The datasets generated and/or analyzed during the current study are available from the corresponding author upon request.
